# FPSIR predicts clinical therapeutic responses and survival outcomes in patients with metastatic colorectal cancer undergoing first-line bevacizumab-containing chemotherapy

**DOI:** 10.3389/fimmu.2026.1683928

**Published:** 2026-01-27

**Authors:** Ya-Nan Li, Feng-Wen Deng, Tian-Qi Lan, Ying Lu, Lin Xiang, Guo-Bin Song, Tian Peng, Xue-Xin Cheng, Hou-Qun Ying

**Affiliations:** 1 Jiangxi Province Key Laboratory of Immunology and Inflammation, The Second Affiliated Hospital, Jiangxi Medical College, Nanchang University, Nanchang, Jiangxi, China; 2 Jiangxi Provincial Clinical Research Center for Laboratory Medicine, The Second Affiliated Hospital, Jiangxi Medical College, Nanchang University, Nanchang, Jiangxi, China; 3 Department of Clinical Laboratory, The Second Affiliated Hospital, Jiangxi Medical College, Nanchang University, Nanchang, Jiangxi, China; 4 Daye People’s Hospital (Affiliated Hospital of Hubei Polytechnic University), Huangshi, Hubei, China; 5 School of Public Health, Jiangxi Medical College, Nanchang University, Nanchang, Jiangxi, China; 6 Department of Laboratory Medicine, Central Hospital of Shangrao City, Shangrao, Jiangxi, China; 7 Shangrao Medical Center of The Second Affiliated Hospital of Nanchang University, Shangrao, Jiangxi, China

**Keywords:** biomarker, colorectal cancer, FPSIR, inflammation, prognosis

## Abstract

**Background:**

Identifying patients who are most likely to benefit from the combination of bevacizumab and chemotherapy (Bev/CT) is essential for the optimal management of metastatic colorectal cancer (mCRC). The aim of this study is to investigate the utility of chronic inflammatory biomarkers in predicting clinical response to Bev/CT and outcomes in patients with mCRC.

**Materials and methods:**

This study enrolled 364 patients with mCRC undergoing first-line Bev/CT therapy. The patients were randomly assigned to discovery (n=249) and validation (n=115) cohorts, maintaining an approximate 2:1 ratio. Two machine learning algorithms, least absolute shrinkage and selection operator (LASSO)-penalized Cox regression and random survival forest (RSF), were employed to identify significant inflammatory biomarkers. Logistic regression, Kaplan-Meier survival analysis, and Cox regression analyses were conducted to evaluate the associations between the clinical outcomes and a product of fibrinogen-pre-albumin ratio and systematic inflammatory ratio (SIR) (FPSIR) and clinical outcomes. The primary endpoints included clinical disease control rate (DCR) and progression-free survival (PFS), 2-year overall survival (OS) was designated as a secondary endpoint.

**Results:**

Following the integration of inflammatory biomarkers identified through the LASSO and RSF algorithms, FPSIR was independently associated with PFS in both the discovery (*p*_log-rank_<0.001, adjusted HR = 1.90, 95%CI=1.40-2.57) and validation cohorts (*p*_log-rank_=0.01, adjusted HR = 1.89, 95%CI=1.21-2.98). Furthermore, FPSIR-H was significantly associated with worse 2-year OS in the two cohorts (discovery cohort: *p*_log-rank_<0.001, adjusted HR = 2.15, 95%CI=1.49-3.10; validation cohort: *p*_log-rank_=0.02, adjusted HR = 1.93, 95%CI=1.06-3.51). Survival nomograms that incorporated CEA, CA19–9 and FPSIR (CCF) score, along with peritoneum metastases, number of metastatic sites, surgical intervention, and treatment regimens could effectively estimate 2-year PFS (AUC = 0.83) and 18-month OS (AUC = 0.71) in the discovery cohort, demonstrating robust performance in the validation cohort (AUC = 0.76 and 0.75 for PFS and OS, respectively). Elevated FPSIR was correlated with diminished DCR in Bev/CT therapy (*p* < 0.01, adjusted OR = 2.24, 95% CI = 1.27-3.96). Serial measurements of FPSIR exhibited dynamic changes that effectively monitored the efficacy of Bev/CT treatment.

**Conclusion:**

Pretreatment FPSIR was identified as a robust biomarker for predicting clinical efficacy and prognosis in mCRC patients receiving first-line Bev/CT, providing a promising strategy to address the long-standing challenge of treatment stratification.

## 1 Introduction

Colorectal cancer represents the fourth leading cause of cancer-related mortality in China, accounting for an estimated 240,000 deaths in 2022 (1). Particularly noteworthy is metastatic colorectal cancer (mCRC), the terminal stage of the disease associated with a particularly dismal prognosis, where only 30-35% of patients achieve 3-year survival (2). The therapeutic landscape has evolved significantly over the past two decades following the emergence of targeted therapies, particularly anti-epidermal growth factor receptor (EGFR) and anti-vascular endothelial growth factor (VEGF) monoclonal antibodies (3), with a 3-year overall survival (OS) rates exceeding 37% in contemporary combination regimens incorporating these biologics (4, 5).

As the current standard first-line therapy, bevacizumab combined with chemotherapy (Bev/CT) has been widely adopted in mCRC management (6). Nevertheless, clinical outcomes remain suboptimal, with an objective response rate of 40-50% and disease control rate of 80-90% observed in patients with treatment of first-line Bev/CT (7–9). More concerning is the subset (10-20%) experiencing progressive disease post-treatment, exhibiting a median OS of 11–14 months and a 3-year survival rate below 20% (10–12). This clinical heterogeneity underscores the need for improved patient stratification strategies. The absence of validated predictive biomarkers for Bev response poses dual challenges. Taking into account the risk of therapeutic failure associated with the cost of monoclonal antibodies, the identification of a method detecting Bev non-responders has become a clinical necessity with economical implications (13). These considerations highlight the imperative for developing robust biomarkers that can reliably distinguish Bev-responsive subpopulations while optimizing healthcase resource utilization.

Previous studies have highlighted biomarkers such as genetic polymorphisms, circulating VEGFA, in the pursuit of predictive factors for mCRC patients undergoing Bev/CT (14). However, population-specific genetic variations fundamentally limit the generalizability of polymorphism-based biomarkers across ethnic groups. While tumor-associated angiogenic factors have undergone rigorous investigation, their predictive utility for prognosis remains inconsistent (15). Moreover, most studies did not address the effectiveness of biomarkers across different treatments or validate them in independent centers; their unsatisfactory efficacy falls short of clinical demands.

Accumulating evidence establishes chronic inflammation as a significant hallmark of mCRC, orchestrating tumorigenesis throughout disease progression (16). Notably, the reprogramming of the inflammatory network within the tumor microenvironment (TME) has emerged as a therapeutically exploitable target, given its dynamic nature and pharmacological reversibility (17, 18). Clinical investigations have strategically leveraged peripheral inflammatory biomarkers, such as the systemic immune-inflammation index (SII), systemic inflammation response rate (SIRI), and systemic inflammation ratios (SIR), as surrogate indicators of TME inflammatory burden (19, 20). However, few of them are widely accepted for mCRC due to inadequate assessment in surgical CRC. Our previous studies illustrated that chronic inflammation represented by fibrinogen-pre-albumin or albumin ratios (FPR or FAR) confer therapeutic resistance to both chemotherapy and tyrosine kinase inhibitors (TKIs) in mCRC and non-small cell lung cancer (21). FPR could relatively quantify sustained chronic inflammatory states, exhibiting strong prognostic correlations in mCRC (22, 23). However, no study has evaluated the role of them and their combined ratios in predicting Bev therapeutic response and progression-free survival (PFS) and OS of mCRC patients undergoing first-line Bev/CT settings.

This study utilized both discovery and validation cohorts to establish and validate six novel inflammatory ratios for prognostic stratification in mCRC. Through the systematic application of two machine-learning algorithms, we identified optimized candidate inflammatory ratios in a discovery cohort. Subsequent validated in an independent cohort. Furthermore, pooled cohort analysis evaluated the predictive capacity of these indices for first-line Bev/CT outcomes, with predefined endpoints including disease control rate (DCR) and survival outcomes.

## 2 Materials and methods

### 2.1 Population

This study complied with the Helsinki Declaration and received approval from the Ethical Committee of the Second Affiliated Hospital of Nanchang University. All eligible cases shall be followed the inclusion criteria: 1) mCRC patients were confirmed through clinical symptoms, imaging detection, circulating tumor biomarker detection, and histopathological detection for those undergoing surgical intervention, and they also had at least one measurable metastatic lesion; 2) the cases exhibited a good performance status (ECOG 0-1) in the hospital from January 2018 to June 2023; 3) all eligible cases received first-line treatment either with Bev in combination with oxaliplatin-based CT (Bev/OX) or Bev combined with irinotecan-based CT (Bev/IRI). Those with acute infection (CRP>10 mg/L), trauma (<6 weeks preoperative), cardiovascular and cerebrovascular disease, liver failure, hematological malignancies, or other solid malignancy were excluded from the present study. The sample size calculation is performed *a priori* using SAS 9.4 software. Based on a two-sided log-rank test with an alpha (α) level of 0.05, a power (1-β) of 0.8, and an assumed hazard ratio (HR) of 2 over an enrolled period of 5 years, the initial calculation indicates that a total of 68 patients shall be required. After accounting for an estimated follow-up loss rate of 15%, the final minimum total sample size is established at 80 participants. All eligible patients are randomly assigned into discovery and validation cohort in an approximate ratio of 2:1.

### 2.2 Baseline characteristics collection and laboratory detection

Demographic and clinicopathological characteristics were systematically recorded from each patient, including imaging features confirmed by computed tomography (CT), magnetic resonance imaging (MRI), single-photon emission computed tomography (SPECT). Laboratory data from whole blood and serum were collected at four clinically defined endpoints: baseline (pre-treatment), post-treatment, the time of the first radiological confirmation of disease control (On-DCR), and the time of the radiological confirmation of progression (On-PD). Sysmex-XN-9000 machine (Sysmex, Tokyo, Japan) was selected to detect the peripheral blood cell counts [white blood cell (WBC), neutrophil (NEU), monocyte (MON), lymphocyte (LYM), platelet (PLT)]. Serum fibrinogen (Fib), plasma albumin and pre-albumin (Alb and pAlb), as well as carcinoembryonic antigen (CEA) and carbohydrate antigen 19-9 (CA19-9), were detected using the Clauss assay, Immunological turbidimetry assays, and chemiluminescence immunoassay, respectively. Quality control procedures ensured both intra-assay and inter-assay coefficients of variation were maintained at <10%. FAR, FPR, SII, SIRI and SIR are defined by the following formulas: FAR=Fib/Alb×100; FPR=Fib/pAlb×1000; SII=(NEU×PLT)/LYM; SIRI= (NEU×MON)/LYM; SIR=(NEU×MON×PLT)/LYM. The six novel inflammatory ratios, namely FPSIR (FPR×SIR), FPSIRI (FPR×SIRI), FPSII (FPR×SII), FASIR (FAR×SIR), FASIRI (FAR×SIRI), FASII (FAR×SII) were calculated based on the formulas outlined in **Supplementary Table S1**. The CCF score was composed of preoperative FPSIR, CEA, and CA19-9, and patients with either none, one or two, and three elevated levels of them (CEA>5ng/ml, CA199>37U/ml, FPSIR>7.7) were considered as 0, 1 and ≥2 score, respectively.

### 2.3 Clinical treatments and response evaluation

Standardized radiological evaluations, including CT, MRI, and SPECT were conducted every 8 weeks according to the protocol to monitor metastatic progression. Tumor biomarker surveillance for CEA and CA19–9 was performed biweekly. Treatment response was classified as complete response (CR), partial response (PR), stable disease (SD), or progressive disease (PD). This assessment was conducted blindly by an independent radiologic review committee following the RECIST v1.1 criteria (24). The DCR represented the proportion of patients exhibiting CR, PR or SD.

### 2.4 Follow-up

The interval times from clinical diagnosis to disease progression, death, or last follow-up are defined as PFS and OS, respectively. DCR and PFS are served as the primary endpoints of our study, while 2-year OS is designated as a secondary endpoint. We conducted follow-ups every three months during the first year, followed by six-month intervals in the second and third years, with a final follow-up deadline was June 2025. Those patients were considered as disease progression with typical imaging of distal metastasis or a persistently increased two-fold of tumor-related biomarkers compared to the most recent measurement (25).

### 2.5 Statistical analysis

In our study, the X-tile 3.6 software (26) is employed to determine the optimal cut-off values for these ratios based on PFS. A Least Absolute Shrinkage and Selection Operator (LASSO)-penalized Cox regression analysis (27) is conducted to select candidate inflammatory ratios, with the penalty parameter (λ) optimized through 10-fold cross-validation using the minimum cross-validation error criterion. Simultaneously, a Random Survival Forests (RSF) learning approach (28) is applied for selecting inflammatory biomarkers, implementing a rigorous multi-seed validation strategy where the algorithm is executed multiple times with varying random seeds (specifically, 123, 1234, and 12345). Ultimately, a Venn diagram facilitated the identification of intersecting biomarkers derives from both LASSO and RSF algorithms to enhance the robustness of variable selection. Continuous variables are summarized using median and inter-quartile range (IQR), while categorical variables are presented as frequencies. The normality of continuous variables is assessed via the Shapiro-Wilk test. Mean is compared using a parametric Student’s t-test when data adhered to normal distribution; otherwise, non-parametric Mann-Whitney test is employed. Categorical variables are compared employing either Chi-squared test or Fisher’s exact tests based on sample size considerations. A logistic regression model is constructed to analyze the relationship between the biomarkers and treatment efficacy, employing odds ratio (OR) along with 95% confidence intervals (CIs) to assess the strength of these associations. A Kaplan-Meier curve with log-rank testing is used to compare PFS and OS across groups. The association between biomarkers and prognosis is assessed by Cox proportional hazards regression analysis, reporting hazard ratio (HR) alongside 95%CIs as measures of association strength. Time-dependent receiver operating characteristic (tdROC) curve are performed to assess the prognosis predictive efficacy of significant serum biomarkers. All statistical analyses are conducted using SPSS 27.0 software (IBM Corp, Armonk, NY, USA) and GraphPad Prism 9.0 software (GraphPad Software, San Diego, CA, USA). LASSO-Cox, RSF, nomogram, and Decision curve analysis (DCA) are performed or plotted using R version 4.4.2 (http://www.r-project.org/), with package dependencies: “glmnet”, “randomForestSRC”, “rms”, “ggDCA”, “rmda”. *P* < 0.05 is considered statistically significant and significance level after Bonferroni-correction is *p*-value=0.017 for multiple tests.

## 3 Results

### 3.1 Clinical characteristics of the eligible patients

**Figure 1:**
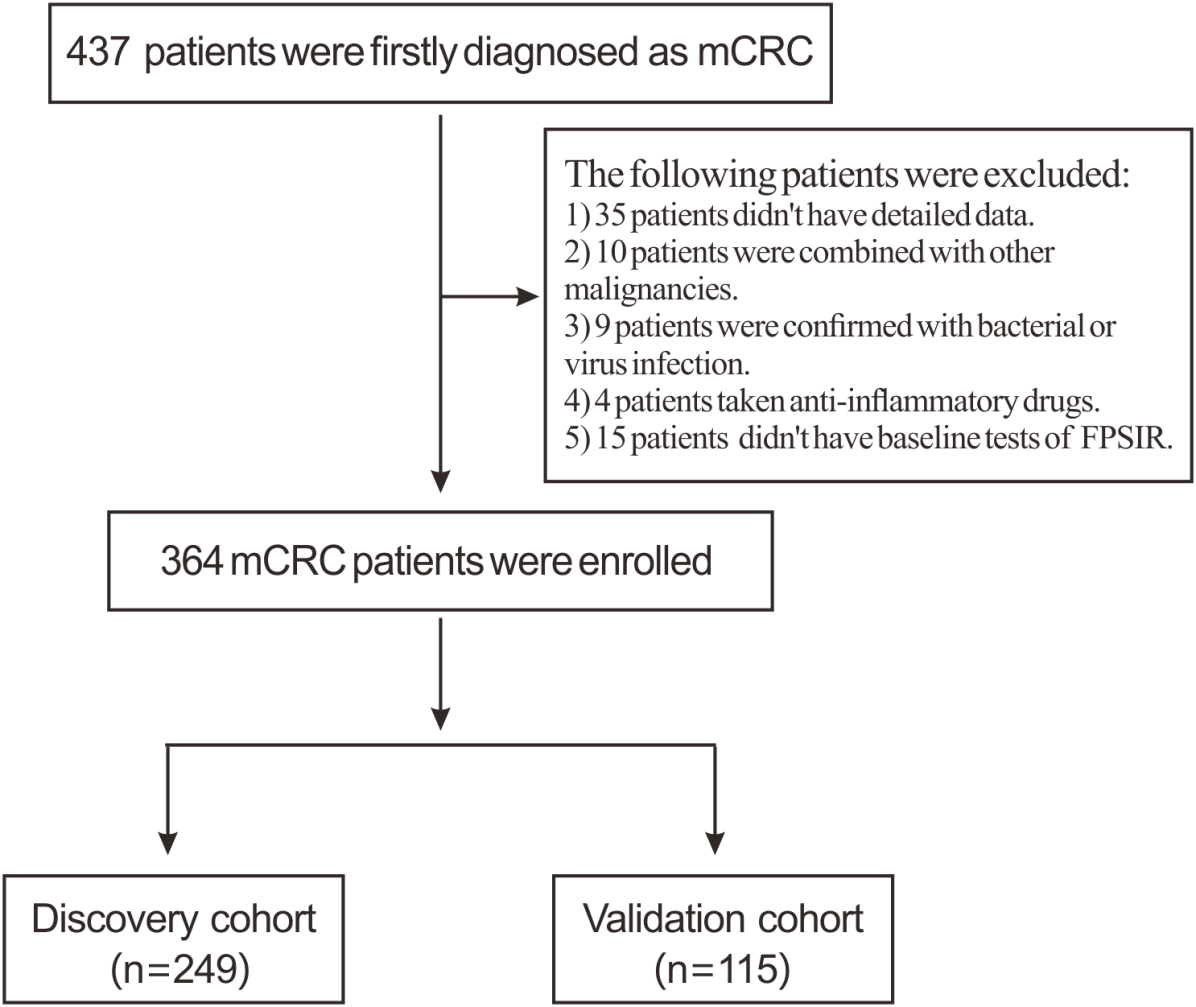
Enrollment flow chart of eligible patients in the present study.

**Figure 2:**
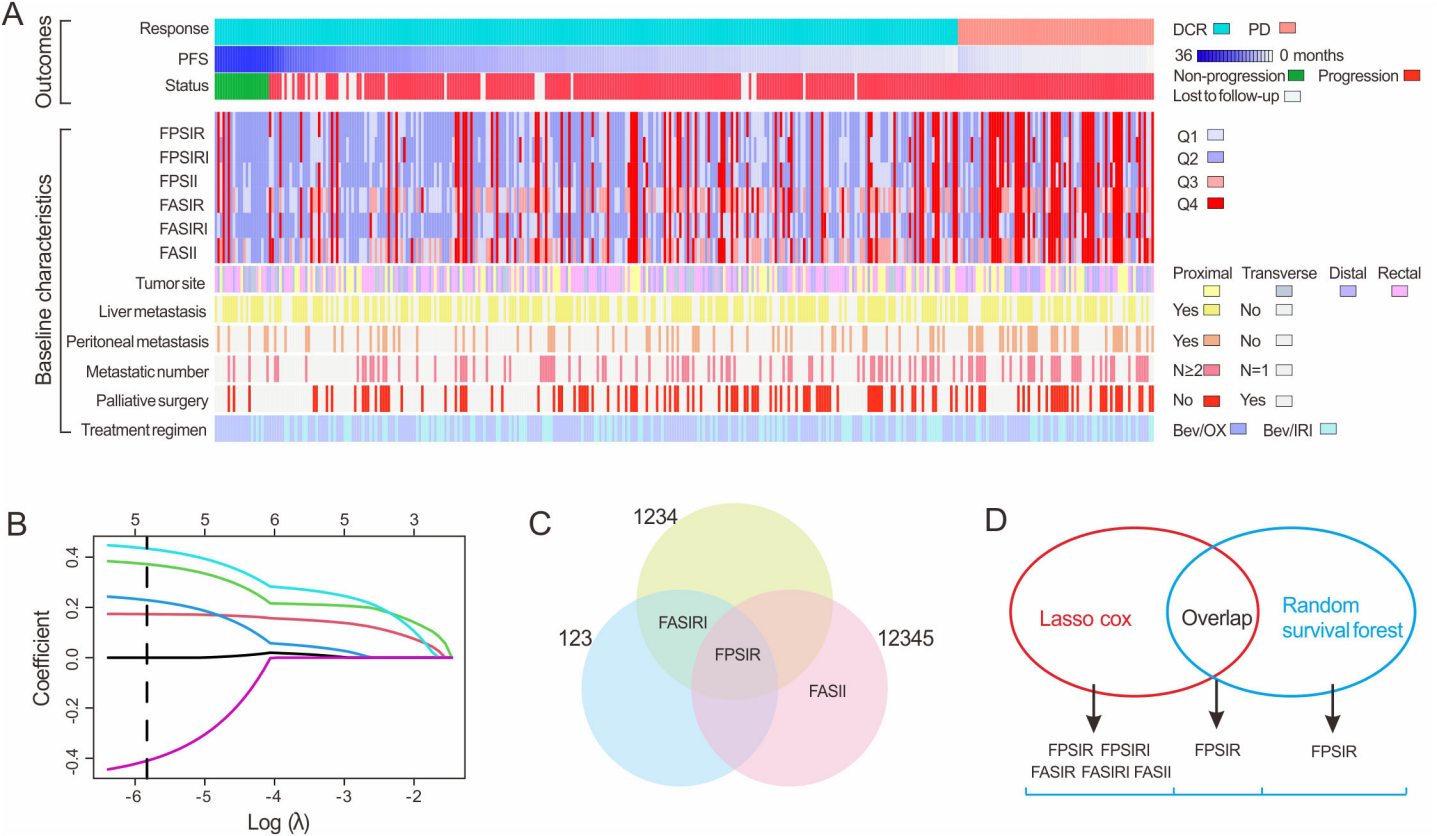
Elevated baseline serum inflammatory markers in patients with PD compared to DCR and biomarker selection using two algorithm. **(A)** Comparisons of baseline laboratory markers, and clinical characteristics; **(B)** LASSO algorithms; **(C)** RSF algorithms; **(D)** cluster analysis of incorporation of markers that were selected from the LASSO or RSF algorithm.

The flowchart of eligible cases is shown in **Figure 1A** total of 364 patients with mCRC were enrolled, with 249 and 115 patients randomly assigned to the discovery and validation cohorts, respectively. The statistical power of both cohorts exceeded 0.8. The characteristics of the patients are summarized in **Table 1** and **Figure 2A**. Within the entire cohort, 131 patients exhibited multisite metastases (≥2 sites), with hepatic metastasis occurring in 62% and peritoneal metastasis in 28%. Two hundreds and forty-three patients received surgical intervention, and these patients were confirmed by pathological detection. The remaining patients were managed non-surgically with systemic therapy alone. In terms of first-line therapy, 242 patients were treated with Bev/OX and 122 with Bev/IRI. After at least two cycles of Bev/CT, treatment response was assessed as follows: one patient achieved CR, 12 had PR, 275 experienced SD, and 76 presented PD, resulting in a DCR of 79%. The median follow-up duration of 364 mCRC patients was 19 months (range, 1 to 36 months). The disease progression was observed in 94% of mCRC patients, with PFS rates at 1- and 2-year being reported as 30% and 10%, respectively. No significant differences were observed regarding clinical characteristics between the discovery and validation cohorts; however, surgical intervention rates, utilization of the Bev/IRI regimen, and PFS outcomes were significantly higher within the validation cohort compared to those in the discovery cohort.

**Table 1:** The baseline characteristics of enrolled mCRC patients in present study.

Characteristics	Discovery cohort	Validation cohort	p-value
	(N = 249)	(N = 115)	
Sex (male)	159 (63.86%)	68 (59.13%)	0.39
Age (>65 years)	64 (25.70%)	38 (33.04%)	0.15
Smoking (yes)	23 (9.24%)	15 (13.04%)	0.27
Drinking (yes)	18 (7.23%)	10 (8.70%)	0.63
Hypertension (yes)	116 (46.59%)	54 (46.96%)	0.95
Diabetes (yes)	42 (16.87%)	19 (16.52%)	0.94
Tumor site			0.39
Proximal colon	65 (26.10%)	26 (22.61%)	
Transverse colon	30 (12.05%)	10 (8.70%)	
Distal colon	64 (25.70%)	27 (23.48%)	
Rectal colon	90 (36.14%)	52 (45.22%)	
Surgical intervention (yes)	156 (62.65%)	87 (75.65%)	0.02
Hepatic metastasis (yes)	159 (63.86%)	68 (59.13%)	0.39
Peritoneal metastasis (yes)	71 (28.51%)	31 (26.96%)	0.76
Number of metastatic site (≥2)	88 (35.34%)	43 (37.39%)	0.71
Treatment regimens			0.04
Bev/OX	174 (69.88%)	68 (59.13%)	
Bev/IRI	75 (30.12%)	47 (40.87%)	
CEA (>5 ng/ml)	168 (67.47%)	74 (64.35%)	0.56
CA19-9 (>37 U/ml)	148 (59.44%)	52 (45.22%)	0.01
SII (>1155)	66 (26.51%)	25 (21.74%)	0.33
NLR (>5.3)	44 (17.67%)	17 (14.78%)	0.49
PLR (>268.3)	50 (20.08%)	23 (20.00%)	0.98
FPSIR (>7.7)	105 (42.17%)	36 (31.30%)	0.05
FPSIRI (>34)	100 (40.16%)	37 (32.17%)	0.14
FPSII (>28.6)	78 (31.33%)	20 (17.39%)	<0.01
FASIR (>55.2)	83 (33.33%)	26 (22.61%)	0.04
FASIRI (>249.2)	58 (23.29%)	23 (20.00%)	0.48
FASII (>93.9)	92 (36.95%)	30 (26.09%)	0.04
Frist reaction (PD)	52 (20.88%)	24 (20.87%)	1.00
PFS (median,IQR,months)	8 (5, 14)	11 (6, 19)	0.06^#^
OS (median,IQR,months)	20 (1, 24)	22 (2, 24)	0.28^#^

^#^compared with the rank-sum test; Abbreviations: mCRC, metastatic colorectal cancer; Bev/OX, Bev combined oxaliplatin-based CT; Bev/IRI, Bev combined irinotecan-based CT; PD, progressive disease; PFS, progression-free survival; OS, overall survival.

### 3.2 Development and evaluation of inflammatory biomarkers

To establish optimal prognostic stratification, X-tile plots were employed to determine cut-off values for nine inflammatory ratios in the discovery cohort (**Supplementary Table S1**). These values were subsequently applied to the independent validation cohort without any re-adjustment. We performed biomarker screening via LASSO regression on the discovery cohort, which identified five candidate biomarkers (FPSIR, FPSIRI, FASIR, FASIRI, FASII; **Figure 2B**). Subsequently, the RSF algorithm, applied with three random seeds (123, 1234, 12345), consistently selected FPSIR as the optimal candidate (**Figure 2C**). An intersection analysis has consequently established FPSIR as the consensus biomarker (**Figure 2D**).

### 3.3 FPSIR and clinicopathological parameters

**Figure 3:**
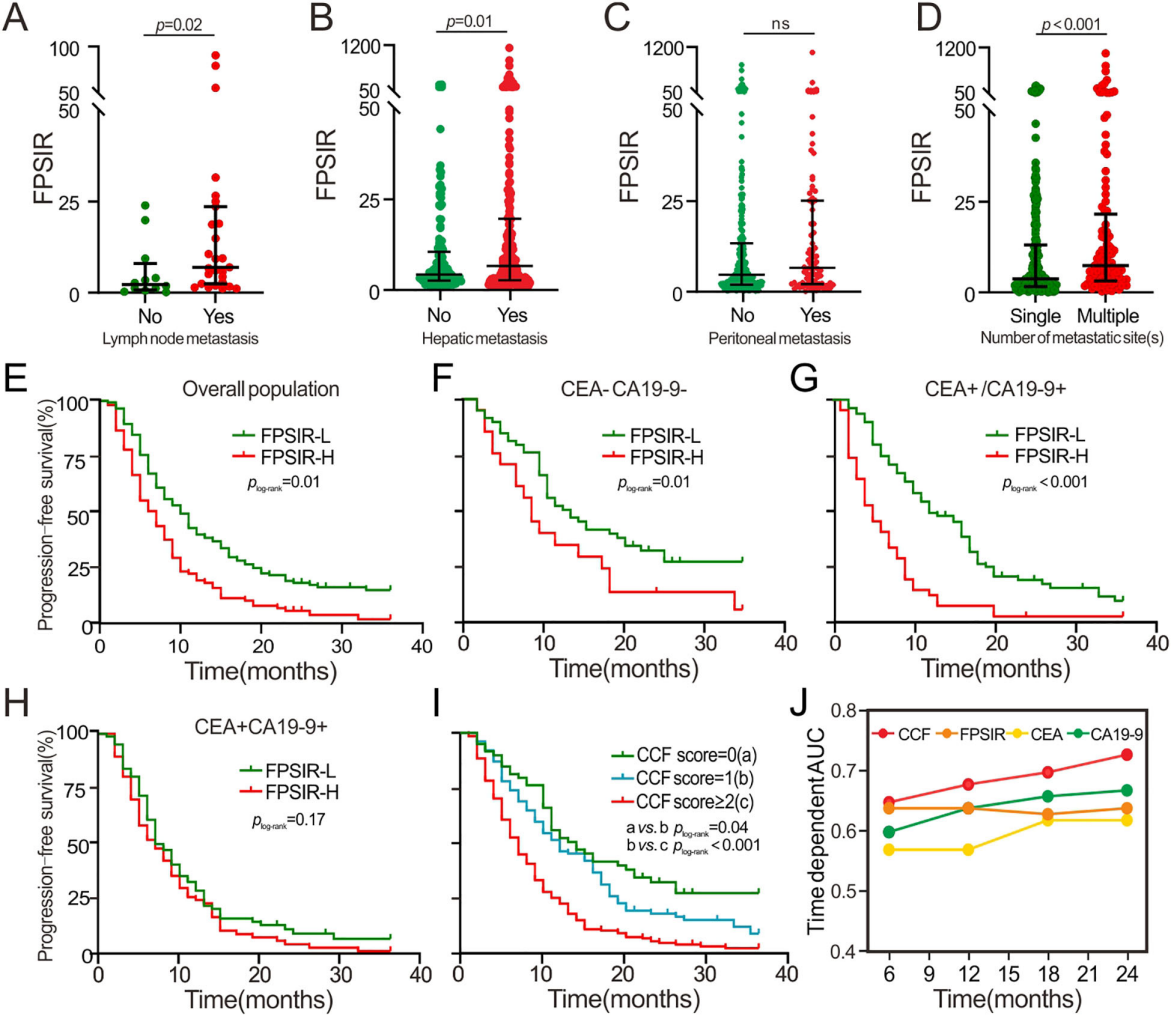
Associations of FPSIR with clinicopathological characteristics and it’s prognostic role across CEA/CA199 subgroups. **(B)** FPSIR comparison between patients with and without hepatic metastasis; **(C)** FPSIR comparison between patients with and without peritoneal metastasis; **(D)** FPSIR comparison between patients with single versus multiple metastatic sites; **(A)** FPSIR comparison between patients with and without lymph node metastasis; **(E–H)** Kaplan-Meier curve of FPSIR-stratified PFS in overall population, both-negative (CEA^-^CA19-9^-^), single-positive (CEA^+-^CA19-9^-+^), and both-positive (CEA^+^CA19-9^+^) subgroup. **(I)** Kaplan-Meier curve of CCF-stratified PFS in overall population; **(J)** time-dependent receiver operating characteristic analysis of CCF, FPSIR, CEA, and CA19–9 in overall population.

Clinically, mCRC patients with lymph node metastasis demonstrated significantly elevated FPSIR levels compared to non-metastatic counterparts (**Figure 3A**). This elevation pattern extended to cases with hepatic/peritoneal metastasis and multisite involvement (≥2 metastatic sites) (**Figures 3B–D**). Given these observed associations, we further stratified the cohort into FPSIR-high (FPSIR-H) and FPSIR-low (FPSIR-L) subgroups. The FPSIR-H subgroup exhibited significantly higher rates of hepatic metastasis and elevated serum CEA and CA19–9 levels compared to the FPSIR-L subgroup (**Supplementary Table S2**).

### 3.4 FPSIR and survival outcomes

In the pooled cohort, a significantly shorter PFS and OS were observed in the FPSIR-H subgroup (n=141) compared to the FPSIR-L patients (n=223, *p*_log-rank_=0.01 for PFS; *p*_log-rank_<0.001 for 2-year OS) (**Supplementary Table S2**; **Figure 3E**; **Supplementary Figure S1A**). As shown in **Table 2**, univariable Cox analysis identified surgical intervention as a significant protective factor for PFS (crude HR = 0.63, 95%CI: 0.48-0.83) within the discovery cohort. Conversely, peritoneal metastasis (crude HR = 1.68, 95%CI: 1.26-2.25), multisite metastasis (crude HR = 1.75, 95%CI: 1.32-2.31), elevated CEA (crude HR = 1.42, 95%CI: 1.06-1.90) and CA19-9 (crude HR = 1.67, 95%CI: 1.26-2.22), along with a high FPSIR (crude HR = 1.80, 95%CI: 1.37-2.38), were identified as significant predictors of poorer PFS. Multivariable Cox analysis, after adjusting for sex, age, smoking, drinking, hypertension, diabetes, tumor site, surgical intervention, number of metastatic sites, and treatment regimens, confirmed that peritoneal metastasis, multisite metastasis, CEA, CA19-9, and FPSIR served as independent prognostic factors. The independent prognostic significance of multisite metastasis (adjusted HR = 1.95, 95%CI: 1.21-3.16), CA19-9 (adjusted HR = 1.69, 95%CI: 1.09-2.62), and FPSIR (adjusted HR = 1.89, 95% CI: 1.21-2.98) was further validated in the validation cohort. Futhermore, FPSIR-H patients had significantly inferior 2-year OS compared to FPSIR-L patients both in the discovery (*p*_log-rank_<0.001, adjusted HR = 2.15, 95% CI: 1.49-3.10) and validation cohorts (*p*_log-rank_=0.02; adjusted HR = 1.93, 95% CI:1.06-3.51) (**Supplementary Table S3**).

**Table 2:** Kaplan-Meier curve and Cox regression analysis of PFS in the discovery and validation cohorts.

Characteristics	Discovery cohort			Validation cohort		
	*p*_log-rank_	Univariate	Multivariate	*p*_log-rank_	Univariate	Multivariate
		HR (95% CI)	HR (95% CI)		HR (95% CI)	HR (95% CI)
Sex(male)	0.30	0.86 (0.65-1.14)	0.83 (0.61-1.13)	0.24	0.78 (0.52-1.18)	0.79 (0.49-1.26)
Age(>65 years)	0.70	0.94 (0.69-1.28)	1.03 (0.72-1.46)	0.69	1.09 (0.72-1.66)	1.09 (0.66-1.81)
Smoking(yes)	0.52	1.16 (0.73-1.84)	1.33 (0.70-2.55)	0.97	0.99 (0.53-1.86)	0.37 (0.08-1.65)
Drinking(yes)	0.60	1.15 (0.68-1.94)	0.93 (0.45-1.93)	0.05	1.99 (0.99-4.00)	5.82 (1.06-31.96
Hypertension(yes)	0.39	1.13 (0.86-1.48)	1.05 (0.76-1.44)	0.33	1.22 (0.82-1.81)	1.04 (0.62-1.73)
Diabetes(yes)	0.12	1.32 (0.93-1.87)	1.37 (0.95-1.97)	0.49	1.20 (0.71-2.04)	1.12 (0.61-2.07)
Tumor site (Proximal colon)	–	–	–	–	–	–
Transverse colon	0.25	0.76 (0.47-1.22)	0.65 (0.40-1.07)	0.78	0.90 (0.41-1.95)	0.75 (0.31-1.80)
Distal colon	0.83	0.96 (0.66-1.40)	0.91 (0.61-1.35)	0.90	1.04 (0.58-1.87)	0.82 (0.43-1.56)
Rectal colon	0.58	0.91 (0.64-1.28)	0.93 (0.64-1.34)	0.97	1.01 (0.60-1.69)	0.80 (0.45-1.42)
Surgical intervention (yes)	<0.01	0.63 (0.48-0.83)	0.63 (0.47-0.84)	0.44	0.83 (0.52-1.33)	0.86 (0.51-1.46)
Hepatic metastasis (yes)	0.85	0.97 (0.73-1.29)	1.03 (0.74-1.43)	0.33	1.22 (0.81-1.83)	1.11 (0.66-1.89)
Peritoneal metastasis (yes)	<0.01*	1.68 (1.26-2.25)	1.57 (1.05-2.34)	0.45	0.84 (0.53-1.32)	0.58 (0.31-1.10)
Number of metastatic sites(≥2)	<0.01	1.75 (1.32-2.31)	1.57 (1.13-2.18)	0.02	1.66 (1.10-2.49)	1.95 (1.21-3.16)
Treatment regimens (Bev/OX)	0.16	1.23 (0.92-1.65)	1.26 (0.92-1.72)	0.37	1.20 (0.80-1.80)	1.24 (0.79-1.95)
CEA(>5ng/ml)	0.02	1.42 (1.06-1.90)	1.55 (1.13-2.12)	0.09	1.46 (0.95-2.24)	1.51 (0.96-2.38)
CA199(>37U/ml)	<0.01*	1.67 (1.26-2.22)	1.63 (1.21-2.20)	<0.01	1.76 (1.17-2.63)	1.69 (1.09-2.62)
FPSIR (>7.7, cut-off value)	<0.01*	1.80 (1.37-2.38)	1.90 (1.40-2.57)	0.01	1.68 (1.11-2.55)	1.89 (1.21-2.98)
FPSIR (Continuous,per SD)	0.02	1.17 (1.03-1.32)	1.13 (1.03-1.25)	0.01	1.24 (1.03-1.49)	1.33 (1.07-1.64)
FPSIR (>5.45, median)	0.03	1.36 (1.04-1.79)	1.50 (1.12-2.01)	<0.01	1.72 (1.15-2.56)	1.88 (1.22-2.90)
FPSIR (>29.4, mean)	0.03	1.49 (1.04-2.13)	1.59 (1.10-2.32)	0.02	1.57 (0.84-2.94)	1.78 (0.91-3.50)
FPSIR (Q1≤2.42)	–	–	–	–	–	–
Q2 (2.42-5.45)	0.07	1.44 (0.97-2.13)	1.64 (1.08-2.47)	0.37	1.28 (0.75-2.18)	1.18 (0.67-2.08)
Q3 (5.45-19.69)	0.07	1.43 (0.97-2.12)	1.66 (1.09-2.51)	0.06	1.67 (0.99-2.81)	1.66 (0.96-2.86)
Q4 (>18.69)	<0.01	1.86 (1.27-2.74)	2.31 (1.51-3.51)	<0.01	2.42 (1.33-4.39)	2.97 (1.57-5.63)
CCF score (0)	–	–	–	–	–	–
1	0.17	1.37 (0.84-2.21)	1.43 (0.87-2.36)	0.09	1.56 (0.86-2.83)	1.41 (0.74-2.68)
≥2	<0.01*	2.58 (1.66-4.00)	2.90 (1.82-4.63)	<0.01	2.36 (1.35-4.12)	2.69 (1.48-4.90)

^*^p < 0.001. HR (95%) was adjusted by sex, age, smoking, drinking, hypertension, diabetes, tumor site, surgical intervention, number of metastatic sites, and treatment regimens. Abbreviations: mCRC, metastatic colorectal cancer; Bev/OX, Bev combined oxaliplatin-based CT. HR, hazard ratio; CI, confidence interval.

To assess the stability of FPSIR, we performed multimodel analyses through four distinct statistical methods: standard deviation (SD), median, mean, and quartile classifications. As illustrated in **Table 2**, multivariate Cox regression identified FPSIR as an independent predictor of PFS in mCRC patients receiving first-line Bev/CT when considering FPSIR as a continuous variable in both the discovery (adjusted HR = 1.13, 95% CI: 1.03-1.25) cohort and the validation (adjusted HR = 1.33, 95% CI: 1.07-1.64) cohort. A consistent association between PFS and FPSIR was also observed when treating it as a dichotomized variable in the discovery (adjusted HR = 1.50, 95% CI = 1.12-2.01 for median cut-off) and in the validation cohort (adjusted HR = 1.88, 95% CI = 1.22-2.90). Furthermore, our quartile-based analysis revealed a dose-response relationship between FPSIR and PFS across its increasing Q2, Q3 and Q4 compared to Q1 within both cohorts (**Table 2**). Additionally, the significant associations were observed between 2-year’ OS and FPSIR with above cut-off values (**Supplementary Table S3**).

### 3.5 FPSIR and prognosis in CEA/CA19-9-defined subgroups

In the subgroup stratified by CEA/CA19-9, FPSIR exhibited its discriminative prognostic capability in both CEA and CA19–9 negative cases (CEA^-^CA19-9^-^) (*p*_log-rank_=0.01 for PFS) (**Figure 3F**; **Supplementary Figure S1B**) and in those with either positive CEA or CA19-9 (CEA^+-^CA19-9^-+^) (*p*_log-rank_<0.001 for both PFS and 2-year OS) (**Figure 3G**; **Supplementary Figure S1C**) under Bonferroni correction (*p ≤* 0.017), However, there is no significant PFS or 2-year OS difference between FPSIR-H and FPSIR-L cases within the positive CEA and CA19-9 (CEA^+^CA19-9^+^) subgroup (**Figure 3H**; **Supplementary Figure S1D**). The median PFS progressively decreased among cases with zero, one, and two scores of CCF (**Figure 3I**), with significant survival differences observed when comparing subgroups with two versus zero scores in the discovery (*p*_log-rank_<0.001, adjusted HR = 2.90, 95%CI: 1.82-4.63) and validation (*p*_log-rank_<0.01, adjusted HR = 2.69, 95%CI: 1.48-4.90) cohorts (**Table 2**). Moreover, CCF achieved a high area under the curve (AUC) for predicting PFS at 6-month (AUC = 0.63), 12-month (AUC = 0.66), 18-month (AUC = 0.68), and 24-month (AUC = 0.71) time-points, outperforming other biomarkers such as CEA, CA19-9, SII, NLR, PLR or FPSIR in the overall population (**Figure 3J**). Consistently, similar predictive performances of CCF and FPSIR were observed for OS (**Supplementary Table S3**; **Supplementary Figures S1E, F**).

### 3.6 Establishment and validation of the PFS prediction model

**Figure 4:**
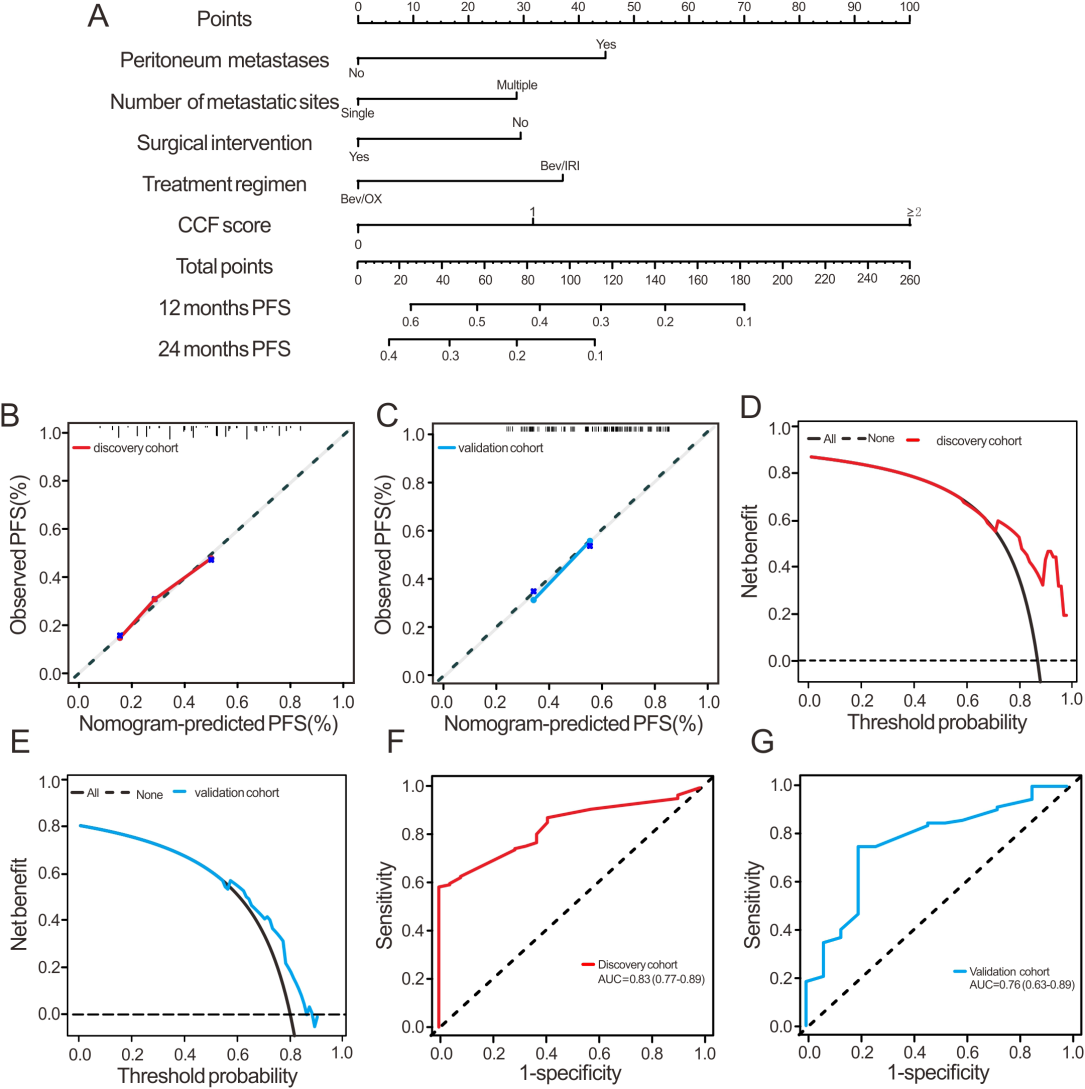
Development and validation of a CCF-incorporated nomogram for one- and two-year PFS prediction in Bev/CT treated mCRC. **(A)** nomogram performance with CCF and clinicopathologic covariates in the discovery cohort; **(B, C)** calibration curves for 2-year PFS prediction in the discovery and validation cohorts; **(D, E)** decision curve analysis demonstrating clinical utility for 2-year PFS in the discovery and validation cohorts; **(F, G)** time-dependent ROC analysis of nomogram-predicted 2-year PFS in the discovery and validation cohorts.

Based on the multivariate analysis of PFS, we developed nomograms to predict 1- and 2-year PFS, and 18-month OS in the discovery cohort. This model incorporates CCF, peritoneal metastasis, number of metastatic sites, surgical intervention, and treatment regimens (**Figure 4A**; **Supplementary Figure S2A**). For predicting 2-year PFS, the model exhibited strong predictive accuracy, with calibration plots showing close agreement between predicted values and observed outcomes in both cohorts (**Figures 4B, C**). Decision curve analysis indicated that the FPSIR model provided a superior net benefit compared to the “treat-none” and “treat-all” strategy at high threshold probabilities, starting from approximately 60% and persisting up to at least 80% in both cohorts (**Figures 4D, E**). The AUC values reached 0.83 for the discovery cohort and 0.76 for the validation cohort (**Figures 4F, G**). Regarding 18-month OS prediction, the model also demonstrated strong accuracy, with calibration plots and decision curve analysis showing similar reliability (**Supplementary Figures S2B–E**). AUCs of OS nomogram model were 0.71 and 0.75 in the discovery and validation cohorts, respectively (**Supplementary Figures S2F, G**). Additional performance metrics for the PFS and OS prediction model, including Brier score, sensitivity, specificity, positive predictive value and negative predictive value, are provided in **Supplementary Table S4**. These model are particularly well-suited for identifying patients at high risk of progression who may benefit from more intensive management strategies.

### 3.7 FPSIR and clinical response

**Figure 5:**
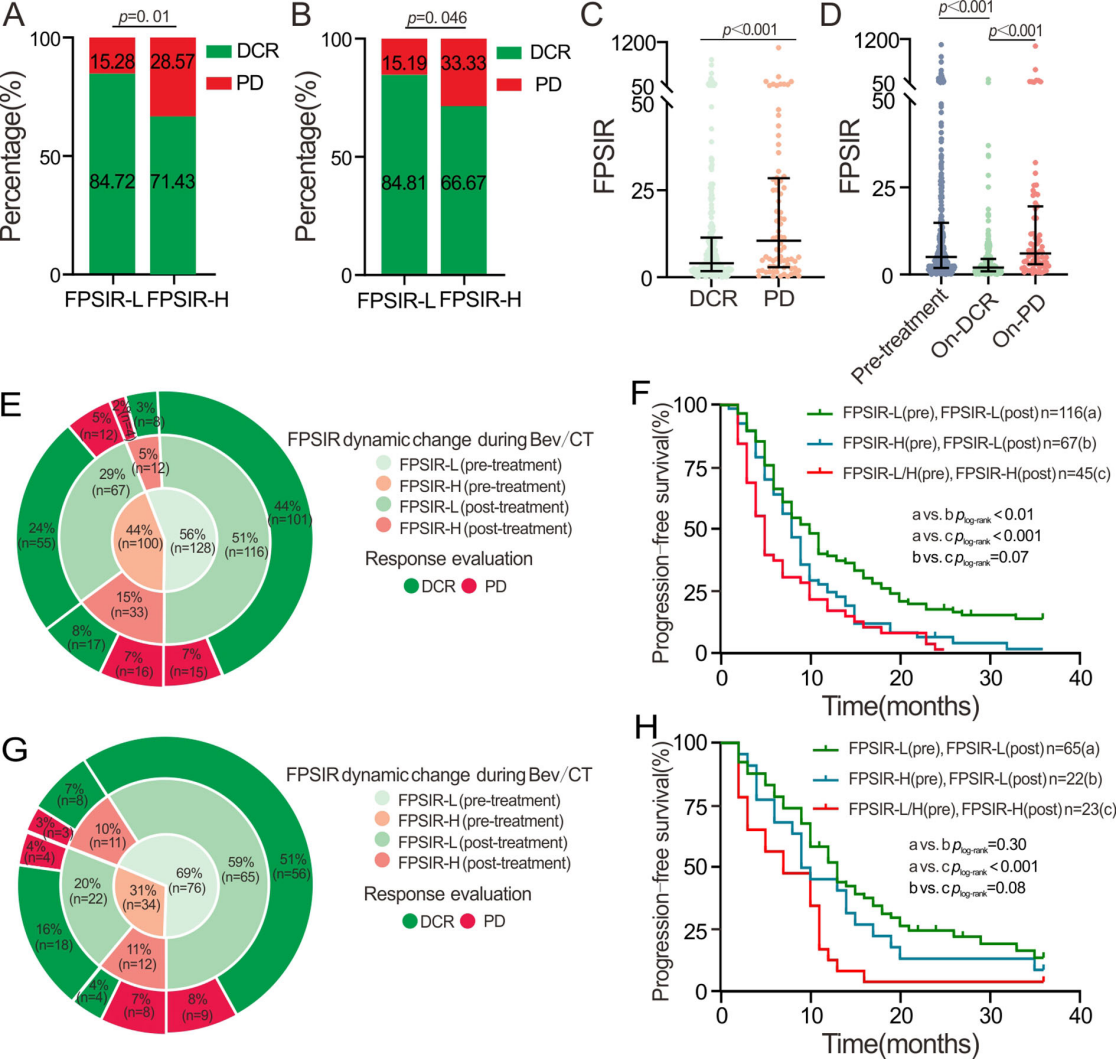
Association of FPSIR with treatment response and dynamic FPSIR changes during Bev/CT treatment for mCRC. **(A, B)** association of FPSIR with DCR in discovery cohort and validation cohorts; **(C)** pre-treatment FPSIR comparison in DCR and PD patients; **(D)** longitudinal FPSIR changes across pre-treatment, on-DCR, on-PD time-points; **(E)** temporal FPSIR dynamics in discovery cohort; **(F)** Kaplan-Meier analysis of FPSIR dynamic change in the discovery cohort; **(G)** temporal FPSIR dynamics in the validation cohort; **(H)** Kaplan-Meier analysis of FPSIR dynamic change in the validation cohort.

According to the clinical response to Bev/CT treatment, patients classified as FPSIR-H exhibited a significantly lower DCR compared to those categorized as FPSIR-L in both the discovery cohort (*p* = 0.01; **Figure 5A**) and the validation cohort (*p* = 0.046; **Figure 5B**). Due to the limited sample size of the FPSIR-H patients with PD response, we combined data from both cohorts. We identified that peritoneal metastasis (adjusted OR = 2.23, 95%CI: 1.08-4.62), multisites metastasis (adjusted OR = 1.68, 95%CI: 1.32-2.13), and high FPSIR (adjusted OR = 2.24, 95%CI: 1.27-3.96) were significantly associated with PD following treatment. Furthermore, these associations with poor treatment responses persisted when considering FPSIR as categorical variables (adjusted OR = 1.45, 95%CI=1.14-1.85 for median cut-off; adjusted OR = 1.55, 95%CI=1.11-2.15 for mean cut-off; adjusted OR = 2.80, 95% CI = 1.25-6.27 for Q4 *vs*. Q1). However, the association was not validated when analyzed as continuous variables (adjusted OR = 1.12, 95%CI: 0.89-1.42 for per SD) (**Table 3**).

**Table 3:** Univariable and multivariable logistic regression analyses of treatment outcomes (PD vs. DCR).

Characteristics	Clinical Bev/CT response		Univariate logistic regression		Multivariate logistic regression	
	DCR,N = 288	PD,N = 76	OR (95% CI)	*p*-value	OR (95% CI)	*p*-value
Sex (male)	115 (39.93%)	22 (28.95%)	0.61 (0.35-1.06)	0.08	0.56 (0.31-1.01)	0.06
Age (>65 years)	82 (28.47%)	20 (26.32%)	0.90 (0.51-1.59)	0.71	0.93 (0.49-1.76)	0.82
Smoking (yes)	28 (9.72%)	10 (13.16%)	1.41 (0.65-3.04)	0.39	1.39 (0.43-4.52)	0.58
Drinking (yes)	20 (6.94%)	8 (10.53%)	1.58 (0.67-3.73)	0.30	1.17 (0.30-4.51)	0.82
Hypertension (yes)	136 (47.22%)	34 (44.74%)	0.91 (0.54-1.50)	0.70	0.83 (0.45-1.53)	0.56
Diabetes (yes)	45 (15.63%)	16 (21.05%)	1.44 (0.76-2.72)	0.26	1.67 (0.83-3.35)	0.15
Tumor site (Proximal colon)	70 (24.31%)	21 (27.63%)	–	–	–	–
Transverse colon	35 (12.15%)	5 (6.58%)	0.48 (0.17-1.37)	0.17	0.42 (0.14-1.26)	0.12
Distal colon	67 (23.26%)	24 (31.58%)	1.19 (0.61-2.34)	0.61	1.19 (0.58-2.43)	0.64
Rectal colon	116 (40.28%)	26 (34.21%)	0.75 (0.39-1.43)	0.38	0.78 (0.39-1.56)	0.48
Surgical intervention (yes)	198 (68.75%)	45 (59.21%)	0.66 (0.39-1.11)	0.12	0.65 (0.37-1.15)	0.14
Hepatic metastasis (yes)	181 (62.85%)	46 (60.53%)	0.91 (0.54-1.52)	0.71	1.16 (0.60-2.23)	0.67
Peritoneal metastasis (yes)	70 (24.31%)	32 (42.11%)	2.27 (1.33-3.84)	<0.01	2.23 (1.08-4.62)	0.03
Number of metastatic sites (≥2)	93 (32.29%)	38 (50.00%)	2.10 (1.26-3.50)	<0.01	1.68 (1.32-2.13)	<0.01
Treatment regimens (Bev/OX)	191 (66.32%)	51 (67.11%)	0.97 (0.56-1.65)	0.90	1.06 (0.59-1.91)	0.84
CEA (>5ng/ml)	190 (65.97%)	52 (68.42%)	1.12 (0.65-1.92)	0.69	1.08 (0.60-1.93)	0.81
CA19-9 (>37U/ml)	150 (52.08%)	50 (65.79%)	1.77 (1.04-3.00)	0.03	1.76 (1.00-3.08)	0.05
FPSIR (>7.7, cut-off value)	99 (34.38%)	42 (55.26%)	2.36 (1.41-3.94)	<0.01*	2.24 (1.27-3.96)	<0.01
FPSIR (Continuous, per SD)	-	-	1.19 (0.95-1.48)	0.13	1.12 (0.89-1.42)	0.34
FPSIR (>5.45, median)	134 (46.53%)	49 (64.47%)	2.09 (1.24-3.52)	<0.01	1.45 (1.14-1.85)	0.04
FPSIR (>29.4, mean)	35 (12.15%)	17 (22.37%)	2.08 (1.09-3.97)	0.03	1.55 (1.11-2.15)	<0.01
FPSIR (Q1≤2.42)	87 (30.21%)	15 (19.74%)	–	-	–	-
Q2 (2.42-5.45)	75 (26.04%)	16 (21.05%)	1.24 (0.57-2.67)	0.59	1.02 (0.44-2.36)	0.96
Q3 (5.45-19.69)	74 (25.69%)	17 (22.37%)	1.33 (0.62-2.85)	0.46	0.99 (0.46-2.29)	0.99
Q4 (>18.69)	52 (18.06%)	28 (36.84%)	3.12 (1.53-6.39)	<0.01	2.80 (1.25-6.27)	0.01

^*^p < 0.001. OR (95%) was adjusted by sex, age, smoking, drinking, hypertension, diabetes, tumor site, surgical intervention, number of metastatic sites, and treatment regimens. Abbreviations: PD, progressive disease; DCR,disease control rate; OR, Odds ratio; CI, confidence interval.

### 3.8 Dynamic monitoring of FPSIR during Bev/CT therapy

In the overall cohort, pre-treatment FPSIR was significantly lower in patients with DCR compared to those with PD (median: 4.05 *vs.* 10.48, *p* < 0.01; **Figure 5C**). Longitudinal analysis revealed a marked reduction in FPSIR from the pre-treatment to the On-DCR time point (median: 5.07 *vs.* 2.03, *p* < 0.001; **Figure 5D**), accompanied by a significant increase in FPSIR at the On-PD time point (median: 6.08, *p* < 0.001; **Figure 5D**) when compared to the On-DCR time point. At follow-up, 51% of patients (116/228) sustained FPSIR-L status, and among these individuals, an impressive 87% (n=101) achieved DCR as clinical outcomes following treatments in the discovery cohort. Notably, 67 patients transitioned from pre-treatment FPSIR-H to post-treatment FPSIR-L status; remarkably, 82% (n=55) exhibited SD to Bev/CT treatment (**Figure 5E**). Conversely, PD was identified in 47 patients after first-line Bev/CT treatment; among them, approximately 60% (n=28) presented with pre-treatment FPSIR-H status (**Figure 5E**). Importantly, within those who progressed to PD status, it was observed that 34% (n=16) maintained elevated FPSIR levels from pre- to post-treatment phases (**Figure 5E**). Furthermore, patients exhibiting post-treatment FPSIR-H had worse PFS than those who transitioned to FPSIR-L during treatment evaluation (mPFS: 5 *vs.* 8 months, *p*_log-rank_=0.07). Notably, those maintaining continuous FPSIR-L (pre- to post-treatment) demonstrated the most favorable outcomes (mPFS: 10 months) (**Figure 5F**). Consistently, 24 patients showed PD as a clinical response to Bev/CT therapy; 50% (n=12) presented baseline FPSIR-H, and 8 patients displayed persistently elevated FPSIR at the On-PD time point (**Figure 5G**) in the validation cohort. A significant proportion (59%, 65/110) consistently maintained FPSIR-L, achieving DCR (86%, 56/65). Moreover, DCR was observed in 82% (18/22) of patients transitioning from pre-treatment FPSIR-H to post-treatment PFSIR-L (**Figure 5G**). The PFS for patients with post-treatment FPSIR-H was significantly inferior to those with continuous FPSIR-L (pre-to-post FPSIR-L) (mPFS: 7 *vs.* 13 months, *p*_log-rank_<0.001). Similarly, the patients who transitioned to FPSIR-L also demonstrated improved PFS (mPFS: 9.5 months) (**Figure 5H**).

## 4 Discussion

In this study, we identified FPSIR as a novel inflammatory biomarker using two independent machine learning algorithms in the discovery cohort. This finding was subsequently validated in a cohort of 115 mCRC patients receiving Bev/CT, thereby confirming its robust prognostic capacity. Our results illustrated that the prognostic nomograms incorporating CCF exhibited superior predictive accuracy for 2-year PFS and 18-month OS, achieving improved predicted efficacy in the discovery (AUC = 0.83 for PFS; 0.71 for OS) and validation (AUC = 0.76 for PFS; 0.75 for OS) cohorts. Furthermore, elevated FPSIR was correlated with diminished DCR in Bev/CT treatment. Notably, serial FPSIR measurements exhibited dynamic changes preceding radiographic progression, which supports its potential utility for real-time therapeutic monitoring.

Previous study showed that sustained VEGF-driven neovascularisation facilitated metastatic dissemination through epithelial-mesenchymal transition (EMT) activation in mCRC (29). This pathogenic mechanism underlies the therapeutic rationale for Bev, a humanized monoclonal antibody targeting VEGF-A isoforms, which has become a cornerstone in mCRC management (30). First-line Bev/CT treatment demonstrates a median PFS of approximately 9 months (6, 31, 32), while there is relatively low PR (33) and approximately 10-20% of patients exhibit primary resistance (7–9), both of which are consistent with our findings. Existing investigations of angiogenic biomarkers such as VEGF-A, VEGF-D, PIGF, and Ang-2 show limited concordance across retrospective cohorts (34, 35), however methodological limitations, including heterogeneous assay platforms, inadequate power for subgroup analyses, and failure to account for temporal biomarker fluctuations, preclude clinical implementation. Emerging evidence positions chronic inflammation as a master regulator of the immunosuppressive TME (36). Transcriptomic analyses demonstrate that inflammatory pathways (JAK/STAT, PI3K/AKT, MAPK and NF-kappaB) coordinately regulate angiogenesis, stromal activation, and chemoresistance through cytokine-mediated crosstalk (particularly IL-6/IL-8 signaling) (37, 38). Unlike transient angiogenic signals, inflammatory indices integrate cumulative microenvironmental alterations, potentially offering enhanced prognostic fidelity. Thus, chronic inflammation can comprehensively reflect the status of TME, which seems more appropriate compared to isolating an angio-genic controversial entity (39).

In this study, we identified six novel inflammatory ratios were significantly elevated in the clinical PD subgroup compared to those in the DCR group. Using LASSO and RSF algorithms, we confirmed that FPSIR serves as the sole unifying feature, demonstrating a significant associated with both PFS and OS. FPSIR has demonstrated significant associations with both regional and distal metastasis, as well as with the metastatic sites of mCRC, illustrating its potential to reflect cancer burden and cancer-derived chronic inflammation. Moreover, it was correlated with PFS and OS irrespective of whether it was represented as continuous or categorical variables across both discovery and validation cohorts, underscoring its reliability as a prognostic biomarker for the disease. The prognostic associations were further validated within the CEA-CA19-9- and CEA+-CA19-9-+ subgroups, indicating that FPSIR serves as an effective complement for predicting unfavorable outcome among patients who derive suboptimal benefits from traditional tumor biomarker classifications. In our study, the predictive efficacy of CEA and CA19–9 alone was found to be insufficient, aligning with the findings reported by *Dirican* et al. (40). In contrast, the CCF score incorporating FPSIR, CEA and CA19–9 markedly enhanced the predictive efficacy for PFS and OS among patients. Additionally, we developed nomograms integrating CCF along with factors such as the peritoneal metastasis status, number of metastatic sites, surgical intervention, and treatment regimen to predict the progression and death risk of individuals. The performance of the nomograms were validated within an independent cohort. Thus, the CCF included nomograms may offer simple, accurate prognosis predictions for patients with mCRC.

Cancer-derived chronic inflammation was involved in reflecting clinical sensitivity, as well as resistance to therapeutic regimens (41). In our study, patients with FPSIR-H exhibited low DCR to first-line Bev/CT across two cohorts. This finding was significantly associated with an unfavorable clinical response to the therapeutic regimen when analyzed as continuous or categorical variables while adjusting for multiple confounding factors. These results indicate that FPSIR serves as a robust chronic inflammatory biomarker for predicting responses to Bev/CT. Our previous studies have demonstrated that cancer-derived inflammation mediates resistance to CT in stage II-III CRC, and its level shows a direct correlation with the magnitude of benefit derived from CT (42). In our cross-sectional and longitudinal follow-up data, we observed that elevated chronic inflammation contributed to unsatisfactory clinical sensitivity to Bev/CT. Furthermore, changes in pre- and post-treatment FPSIR within the longitudinal cohort suggest that serial testing of FPSIR can effectively monitor the efficacy of Bev/CT and predict patient prognosis during Bev/CT treatment.

Several limitations of this study should be considered. First, the fact that both cohorts originated from a single Chinese center and involved a small sample size may restrict the generalizability of our findings. Second, while a cut-off value of 7.7 for FPSIR has been established and validated in an independent cohort, its broader applicability requires further confirmation in external populations. Third, Bev is recommended for first-line targeted therapy in mCRC patients which does not consider the status of molecular detection in clinic. The majority of the patients were not detected the status of MSI/dMMR and *RAS/BRAF* mutation, and there was no side-effects data of the treatment, it maybe an another limitation for us to comprehensive assessment of their influence on survival outcome, clinical response and side-effects of the treatment. Consequently, external validation in multi-center, ethnically diverse populations and in cohorts treated with other targeted therapeutic regimens are warrant. Fourth, the study prioritized DCR and PFS over OS as primary endpoints. This decision is consistent with the objective of identifying biomarkers for Bev-based therapy, where disease stabilization is a crucial treatment benefit. Additionally, a high censoring rate (>20%) in the 3-year OS data currently hinders a definitive prognostic analysis. Finally, the precise biological mechanisms by which FPSIR is associated with chronic inflammation and patient outcomes remain to be fully elucidated.

In conclusion, FPSIR represents a promising and robust biomarker for predicting the response to Bev/CT, monitoring disease progression, and predicting prognosis in patients with mCRC undergoing Bev/CT treatment. Prognostic nomograms that incorporates CCF may facilitate the prediction of individual progression and death risk, thereby aiding clinicians in the management of mCRC patients.

## Data Availability

The raw data supporting the conclusions of this article will be made available by the authors, without undue reservation.

## Supplementary material

Supplementary Figure 1 Associations of FPSIR with 2-year OS in the overall population and subgroups stratified by status of CEA and CA19–9 as well as CCF score. **(A–E)** Kaplan-Meier curves of 2−year OS stratified by FPSIR in the overall population, CEA^−^CA19−9^−^, CEA^+−^CA19−9^−+^, CEA^+^CA19−9^+^, and CCF subgroups. **(F)** time-dependent ROC predicts 18-months OS.

Supplementary Figure 2 Development and validation of a CCF-incorporated nomogram for 18-month OS prediction in Bev/CT treated mCRC. **(A)** nomogram performance with CCF and clinicopathologic covariates in the discovery cohort; **(B, C)** calibration curves for 18-month OS prediction in the discovery and validation cohorts; **(D, E)** decision curve analysis demonstrating clinical utility for 18-month OS in the discovery and validation cohorts; (**F, G)** time-dependent ROC analysis of nomogram-predicted 18-month OS in the discovery and validation cohorts.
