# Supplementary material for: FPSIR predicts clinical therapeutic responses and survival outcomes in patients with metastatic colorectal cancer undergoing first-line bevacizumab-containing chemotherapy
Source: Front Immunol. 2026 Jan 27;17:1683928. doi: 10.3389/fimmu.2026.1683928 (PMC12886467; doi:10.3389/fimmu.2026.1683928)
Supplement: Supplementary Figure 1 — Associations of FPSIR with 2-year OS in the overall population and subgroups stratified by status of CEA and CA19–9 as well as CCF score. (A–E) Kaplan-Meier curves of 2−year OS stratified by FPSIR in the overall population, CEA−CA19−9−, CEA+−CA19−9−+, CEA+CA19−9+, and CCF subgroups. (F) time-dependent ROC predicts 18-months OS. [file DataSheet1.doc]

**Supplementary Materials**

**Figure S1**


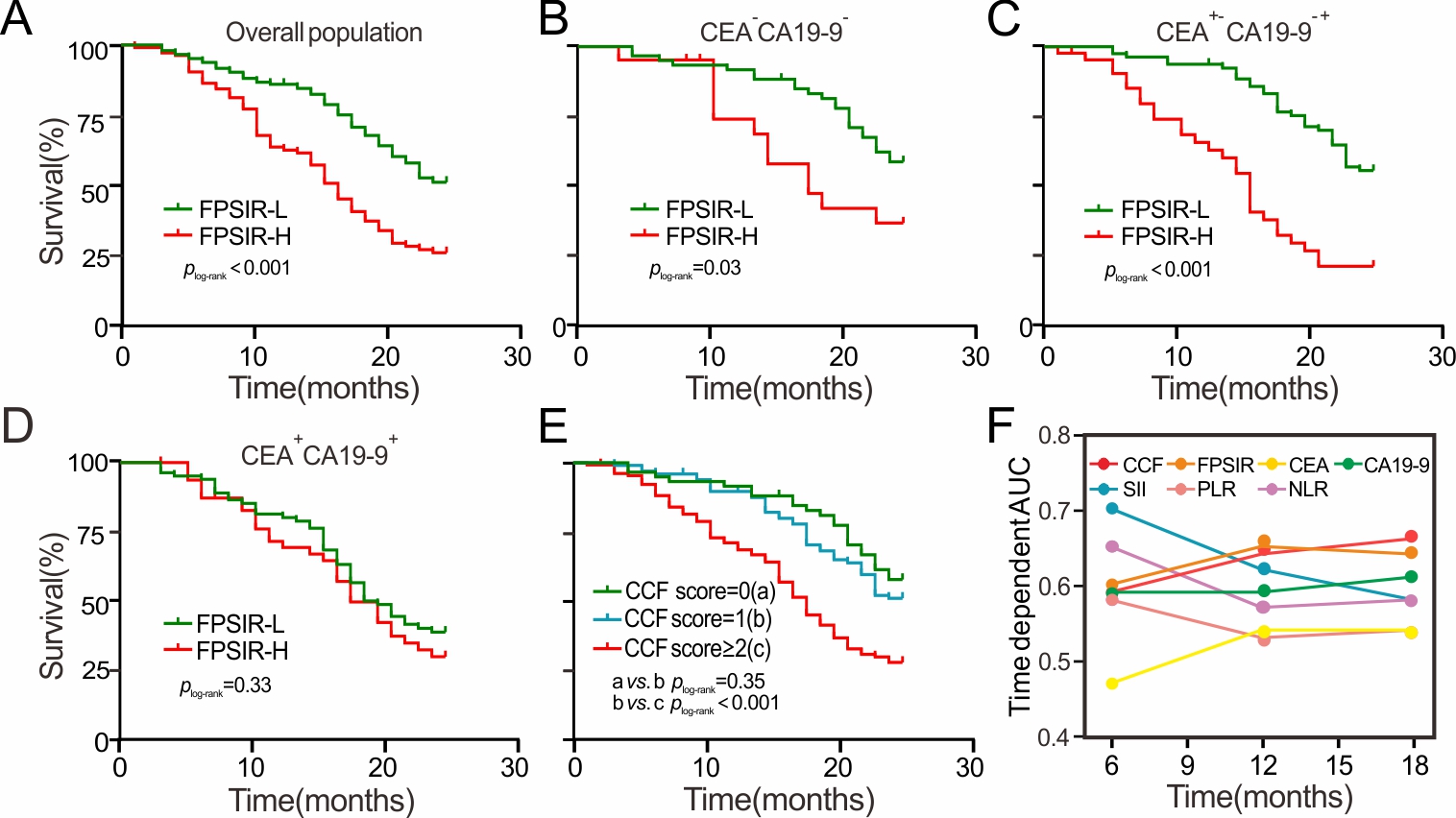


**Figure S2**


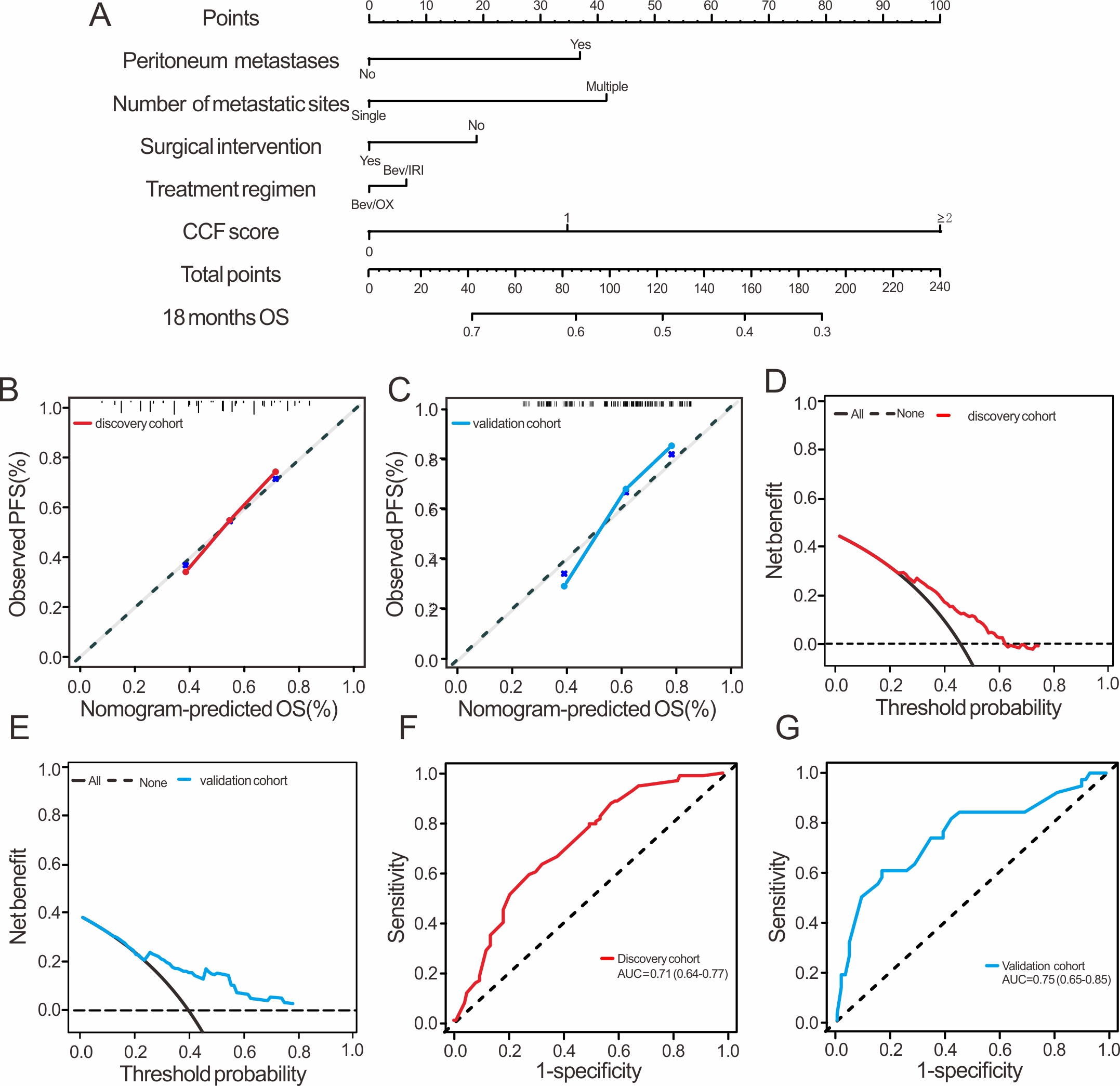


Table S1. The definition and cut-off values of the inflammatory ratios in the present study.

| **Ratios** | **Calculation formulas** | **Cut-off values** |
| --- | --- | --- |
| FPR | Fibrinogen/pre-albumin×1000 | - |
| FAR | Fibrinogen/Albumin×100 | - |
| SIR | (Neutrophils × Monocytes × Platelets)/Lymphocytes | - |
| SIRI | (Neutrophils × Monocytes)/Lymphocytes | - |
| SII | (Neutrophils × Platelets)/Lymphocytes | 1155 |
| FPSIR | FPR×SIR | 7.7 |
| FPSIRI | FPR×SIRI | 34 |
| FPSII | FPR×SII | 28.6 |
| FASIR | FAR×SIR | 55.2 |
| FASIRI | FAR×SIRI | 249.2 |
| FASII | FAR×SII | 93.9 |
| NLR | Neutrophils/Lymphocytes | 5.3 |
| PLR | Platelets/Lymphocytes | 268.3 |

**Notes:** the cut-off values of the ratios are calculated using X-tile software according to progression-free survival.

**Table S2** Clinicopathological characteristics of mCRC patients stratified by FPSIR levels in the overall cohort.

| **Characteristics** | **FPSIR-L** | **FPSIR-H** | ***p*-value** |
| --- | --- | --- | --- |
|  | **(N =223)** | **( N = 141)** |  |
| Tumor site |  |  | 0.06 |
| Proximal colon | 61 (27.35%) | 30 (21.28%) |  |
| Transverse colon | 17 (7.62%) | 23 (16.31%) |  |
| Distal colon | 55 (24.66%) | 36 (25.53%) |  |
| Rectal colon | 90 (40.36%) | 52 (36.88%) |  |
| Hepatic metastasis (yes) | 129 (57.85%) | 98 (69.50%) | 0.03 |
| Peritoneal metastasis (yes) | 57 (25.56%) | 45 (31.91%) | 0.19 |
| Number of metastatic site (≥2) | 72 (32.29%) | 59 (41.84%) | 0.06 |
| CEA (>5 ng/ml) | 136 (60.99%) | 106 (75.18%) | <0.01^*^ |
| CA19-9 (>37 U/ml) | 109 (48.88%) | 91 (64.54%) | <0.01^*^ |
| Frist reaction (PD) | 34 (15.25%) | 42 (29.79%) | <0.01^*^ |
| PFS (median,IQR,months) | 11 (6, 19) | 7 (4, 11) | 0.01^#^ |
| OS (median,IQR,months) | 22 (3, 24) | 16 (1, 24) | <0.01^*^ |

**Note:** ^#^compared with the log-rank test. ^*^*p* < 0.001. **Abbreviations:** mCRC, metastatic colorectal cancer; FPSIR-L, low FPSIR level; FPSIR-H, high FPSIR level; PD, progressive disease; PFS, progression-free survival; OS, overall survival.

Table S3. Kaplan-Meier curve and Cox regression analysis of 2-year OS in the discovery and validation cohorts.

| **Characteristics** | **Discovery cohort** | | | **Validation cohort** | | |
| --- | --- | --- | --- | --- | --- | --- |
|  | ***p*_log-rank_** | **Univariate** | **Multivariate** | ***p*_log-rank_** | **Univariate** | **Multivariate** |
|  |  | **HR (95% CI)** | **HR (95% CI)** |  | **HR (95% CI)** | **HR (95% CI)** |
| Sex(male) | 0.58 | 0.91 (0.64-1.28) | 0.82 (0.57-1.19) | 0.42 | 0.81 (0.48-1.35) | 0.85 (0.48-1.52) |
| Age(>65 years) | 0.58 | 0.89 (0.60-1.32) | 0.92 (0.60-1.42) | 0.61 | 0.87 (0.51-1.50) | 1.07 (0.56-2.02) |
| Smoking(yes) | 0.98 | 1.01 (0.56-1.82) | 1.17 (0.47-2.93) | 0.68 | 0.85 (0.39-1.86) | 0.90 (0.14-1.73) |
| Drinking(yes) | 0.98 | 0.99 (0.52-1.89) | 0.98 (0.36-2.67) | 0.12 | 1.86 (0.84-4.09) | 6.89(1.31-28.44) |
| Hypertension(yes) | 0.88 | 0.97(0.70-1.36） | 0.96 (0.65-1.42) | 0.29 | 1.31 (0.79-2.17) | 1.08 (0.59-1.98) |
| Diabetes(yes) | 0.67 | 1.10 (0.72-1.68) | 1.13 (0.72-1.76) | 0.85 | 0.94 (0.46-1.90) | 0.99 (0.46-2.18) |
| Tumor site (Proximal colon) | - | - | - | - | - | - |
| Transverse colon | 0.41 | 0.77 (0.41-1.43) | 0.72 (0.38-1.35) | 0.43 | 1.50 (0.55-4.05) | 1.35 (0.43-4.20) |
| Distal colon | 0.86 | 0.96 (0.60-1.52) | 0.99 (0.61-1.59) | 0.44 | 1.36 (0.63-2.96) | 1.15 (0.49-2.69) |
| Rectal colon | 0.65 | 0.91 (0.59-1.39) | 0.89 (0.57-1.39) | 0.33 | 1.41 (0.70-2.82) | 1.58 (0.65-2.81) |
| Surgical intervention (yes) | 0.05 | 0.71 (0.50-1.00) | 0.76 (0.53-1.09) | 0.83 | 0.94 (0.52-1.70) | 0.86 (0.45-1.62) |
| Hepatic metastasis (yes) | 0.45 | 0.88 (0.62-1.24) | 0.78 (0.54-1.12) | 0.11 | 1.54 (0.90-2.63) | 1.72 (0.96-3.09) |
| Peritoneal metastasis (yes) | 0.01 | 1.57 (1.20-2.24) | 1.38 (0.90-2.12) | 0.46 | 0.79 (0.43-1.46) | 0.53 (0.27-1.07) |
| Number of metastatic sites(≥2) | <0.01* | 1.79 (1.28-2.52) | 1.80 (1.26-2.58) | 0.04 (1.10-2.49) | 1.70 (1.02-2.82) | 1.39 (0.79-2.43) |
| Treatment regimens (Bev/OX) | 0.76 | 0.94 (0.65-1.37) | 0.98 (0.67-1.44) | 0.63 (0.80-1.80) | 1.13 (0.68-1.88) | 1.07 (0.60-1.88) |
| CEA (>5ng/ml) | 0.27 | 1.23 (0.85-1.77) | 1.11 (0.75-1.63) | 0.10 | 1.58 (0.91-2.75) | 1.64 (0.92-2.92) |
| CA199 (>37U/ml) | 0.02 | 1.67 (1.09-2.18) | 1.42 (0.99-2.03) | <0.01 | 2.15 (1.29-3.59) | 2.19 (1.26-3.81) |
| FPSIR (>7.7, cut-off value) | <0.01* | 2.21 (1.57-3.10) | 2.15 (1.49-3.10) | 0.02 | 1.86 (1.10-3.12) | 1.93 (1.06-3.51) |
| FPSIR (Continuous,per SD) | 0.15 | 1.15 (0.96-1.35) | 1.05 (0.87-1.27) | 0.057 | 1.23 (0.99-1.53) | 1.27 (0.99-1.61) |
| FPSIR (>5.45, median) | <0.01* | 1.91 (1.36-2.68) | 1.83 (1.25-2.68) | 0.01 | 1.90 (1.14-3.14) | 1.91 (1.08-3.37) |
| FPSIR (>29.4, mean) | <0.01 | 1.82 (1.19-2.77) | 1.69 (1.07-2.65) | 0.41 | 1.43 (0.61-3.32) | 1.33 (0.53-3.34) |
| FPSIR (Q1≤2.42) | - | - | - | - | - | - |
| Q2 (2.42-5.45) | 0.04 | 1.71 (1.01-2.89) | 1.64 (0.94-2.84) | 0.902 | 1.05 (0.52-2.12) | 1.01 (0.48-2.12) |
| Q3 (5.45-19.69) | 0.02 | 2.24 (1.33-3.76) | 2.14 (1.21-3.78) | 0.039 | 1.96 (1.04-3.71) | 1.80 (0.90-3.58) |
| Q4 (>18.69) | <0.01* | 2.84 (1.70-4.75) | 2.89 (1.64-5.09) | 0.124 | 1.83 (0.85-3.93) | 2.27 (0.95-5.44) |
| CCF score (0) | - | - | - | - | - | - |
| 1 | 0.28 | 1.43 (0.75-2.72) | 1.41 (0.73-2.71) | 0.938 | 0.97 (0.43-2.16) | 0.94 (0.41-2.16) |
| ≥2 | <0.01* | 2.57 (1.62-4.08) | 2.305(1.259-4.219) | 0.011 | 2.394(1.217-4.711) | 2.748(1.344-5.619) |
| ≥2 | <0.01 | 2.53 (1.41-4.53) | 2.31 (1.26-4.22) | 0.011 | 2.39 (1.22-4.71) | 2.75 (1.34-5.62) |

**Note:** ^*^*p* < 0.001. HR (95%) was adjusted by sex, age, smoking, drinking, hypertension, diabetes, tumor site, surgical intervention, number of metastatic sites, and treatment regimens. **Abbreviations:** mCRC, metastatic colorectal cancer; Bev/OX, Bev combined oxaliplatin-based CT. HR, hazard ratio; CI, confidence interval.

Table S4. Model performance in the discovery and validation cohorts.

| **Model parameters** | **Discovery cohort** | **Validation cohort** |
| --- | --- | --- |
| PFS Prediction Model |  |  |
| Brier Score (%) | 0.12 | 0.15 |
| Sensitivity (%) | 59 | 53 |
| Specificity (%) | 83 | 70 |
| Positive Predictive Value (%) | 95 | 88 |
| Negative Predictive Value (%) | 29 | 27 |
| 18-Month OS Prediction Model |  |  |
| Brier Score (%) | 0.22 | 0.21 |
| Sensitivity (%) | 58 | 48 |
| Specificity (%) | 69 | 73 |
| Positive Predictive Value (%) | 58 | 50 |
| Negative Predictive Value (%) | 69 | 71 |
